# Current Architectural and Developmental Approaches in Artificial Intelligence Models for Prostate Cancer Detection and Management: A Technical Report

**DOI:** 10.7759/cureus.81748

**Published:** 2025-04-05

**Authors:** Kian A Huang, Haris K Choudhary, Kyoung A V Lee, Corey D Tesdahl, Paul C Kuo

**Affiliations:** 1 General Surgery, University of South Florida Health Morsani College of Medicine, Tampa, USA

**Keywords:** computer vision, convolutional neural network, prostate cancer, prostate cancer detection, prostate cancer treatment

## Abstract

Prostate cancer is a prevalent malignancy among men and remains a major cause of cancer-related mortality. The increasing incidence of cases underscores the need for advancements in diagnostic methodologies. Artificial intelligence (AI) is emerging as a transformative tool in addressing challenges in prostate cancer diagnostics, particularly in the analysis of histopathological whole-slide images and the refinement of algorithmic Gleason grading. Traditional diagnostic approaches, including the Gleason grading system and prostate-specific antigen (PSA) testing, are subject to variability and inefficiencies, placing a significant burden on pathologists and potentially delaying accurate diagnoses. This report explores the role of AI-driven models, such as convolutional neural networks and clinically validated deep learning systems, in enhancing diagnostic accuracy for tumor detection and Gleason grading. These models incorporate advanced techniques, including ensemble learning, specialized pooling mechanisms, and semi-supervised learning, to improve efficiency in feature extraction. Additionally, AI models integrating PSA data have demonstrated improved accuracy in risk stratification, reducing the reliance on traditional PSA thresholds and minimizing unnecessary biopsies. However, challenges persist, such as inconsistencies in data sources, imaging domain shifts, and the absence of standardized stain normalization, which hinder AI’s widespread clinical adoption. By examining the current technological landscape, this report highlights AI’s potential to revolutionize prostate cancer diagnostics, enhancing workflow efficiency and diagnostic precision in clinical practice.

## Introduction

Prostate cancer is the second most commonly diagnosed cancer in men and the fifth leading cause of cancer-related deaths worldwide, accounting for approximately 15% of all cancers globally [1,2]. It is the most prevalent cancer in men across 112 countries, with the average age of presentation being 66 years; the number of new annual prostate cancer cases is projected to increase from 1.4 million in 2020 to 2.9 million by 2040 [1,2]. Screening for prostate cancer traditionally involves biopsy and analysis of hematoxylin and eosin (H&E)-stained prostate tissue sections, with pathologists assigning Gleason scores (GS) to evaluate cancer severity and inform treatment decisions. This process requires approximately 12 biopsies per patient, generating millions of samples annually and placing a substantial workload on pathologists worldwide [3].

While pathologists’ biopsy readings of H&E tissue samples remain the cornerstone of diagnosis, emerging artificial intelligence (AI) models have demonstrated success using additional modalities such as prostate-specific antigen (PSA) and magnetic resonance imaging (MRI). These advancements highlight AI’s potential to provide a multifaceted approach to screening and diagnosing prostate cancer. Further, the potential to identify prostate cancer with MRI using AI-assisted methods may increase incidental and serendipitous early detection in patients undergoing MRI for other conditions. The increasing demand for prostate cancer screening and the risk of pathologist burnout underscore the need for AI-driven solutions. Neural models have shown promise in assisting with biopsy analysis, malignancy detection, prognosis, risk stratification, and treatment planning [3].

This report consolidates the current understanding of AI applications in prostate cancer detection and management, details key strategies and methods, and addresses limitations while introducing the fundamental concepts of deep learning model training to enhance physician knowledge and application.

## Technical report

Gleason scoring using AI-driven systems

AI has emerged as a transformative tool in pathology, especially for diagnosing and grading prostate cancer via histopathological biopsies. Prostate cancer grading heavily relies on the Gleason grading system that assesses the architectural patterns of tumor cells to predict cancer aggressiveness and potential for metastasis. The GS is derived by grading the two most common patterns in a biopsy, scored from 1 (most differentiated) to 5 (least differentiated), and then adding them together. However, a GS below 6 is rarely reported. However, GS determination can be subjective due to variability in pathologist interpretation, leading to significant interobserver and intraobserver variability [4]. This subjectivity can result in inconsistent diagnoses and treatment recommendations. AI-driven systems using digital whole-slide images (WSIs) aim to reduce these inconsistencies and enhance diagnostic precision by providing quantitative and standardized evaluations [5,6]. Among these systems, models such as the fully convolutional network (FCN) with U-network (U-Net) architecture and residual network extension with 50 layers (ResNeXt50), the clinically validated DeepDx® Prostate and entries from the Prostate cANcer graDe Assessment (PANDA) challenge highlight the potential of AI in pathology. Table 1 contains a variety of useful definitions of terminology used during AI model development and training.

**Table 1:** Key AI concepts relevant to prostate cancer models. This table provides an overview of essential AI concepts and methodologies used in prostate cancer modeling. It includes various machine learning and deep learning techniques, training strategies, model evaluation approaches, and data handling methods that contribute to improving predictive accuracy and robustness in prostate cancer management. AI: artificial intelligence

Concept	Description
Machine learning models	Algorithms that learn patterns from data to make predictions or decisions without explicit programming
Supervised learning	Training models on labeled data, where each input has a corresponding output label
Semi-supervised learning	Combines labeled and unlabeled data to improve model performance
Weakly supervised learning	Learning from limited or noisy labeled data, often using approximate labels or partial supervision
Unsupervised learning	Learning patterns and structures from unlabeled data without predefined labels
Self-supervised learning	Training models to predict parts of the data from other parts, generating pseudo-labels from unlabeled data
Active learning	Training strategy where the model selectively queries the most informative data points for labeling
Multiple-instance learning	Learning framework where labels are assigned to groups (bags) of instances, not individual instances
Transfer learning	Leveraging pre-trained models from related tasks to improve performance on a new task with limited data
Deep learning models	Subset of machine learning that uses multilayered neural networks to model complex patterns in data
Convolutional neural network models	Specialized neural networks designed to process grid-structured data such as images
Patch-based approaches	Dividing images into smaller patches for individual analysis
End-to-end training	Training approach where a model learns all components jointly from input to output, without intermediate steps
Consensus-based training	Combining predictions from multiple models to improve robustness and reduce biases
Domain-agnostic feature learning	Learning features that are not specific to a particular domain, allowing for broader applicability
Ensemble methods	Combining multiple models to improve predictive accuracy and reduce variance
Data augmentation	Techniques to artificially increase the dataset size by creating modified versions of existing data
Hyperparameter optimization	The process of tuning model parameters that are not learned during training to enhance performance
Cross-validation	A resampling method to evaluate model performance by splitting data into training and testing subsets
Random oversampling	Balancing class distributions by duplicating minority class samples in the training data
Uncertainty-based sampling	Selecting data points for labeling where the model has the highest uncertainty in predictions
Pre-labeling	Assigning preliminary labels to data, often as a starting point for further refinement
Area under the receiving operating characteristic curve	Performance metric evaluating the ability of a model to distinguish between classes, especially in classification tasks

AI algorithms for Gleason scoring typically follow a multistep pipeline that begins with the digitization of histopathological slides into high-resolution WSIs. These images are then preprocessed to ensure consistency across different laboratory conditions, reducing the batch effect. Data manipulation techniques are part of this preprocessing step. One example of these techniques is stain normalization, which is critical for reducing domain shifts caused by differences in staining protocols across institutions. This technique ensures that the model performs consistently, regardless of how or where the slides were prepared. Data augmentation, a technique that creates new data from existing data by rotations, flips, and random cropping, helps to enhance model robustness by simulating variations in slide presentation [5,7].

Following the preprocessing step, image-based analysis using AI can use algorithms that are well-suited for image-based analyses, such as convolutional neural networks (CNNs) or transformer-based architectures. After designing the algorithm and specifying specific parameters for training, these algorithms are “trained” to think like a diagnostician on a subset of data that is usually labeled with information that the algorithm should learn. These features may include different glandular morphologies and their labels, as well as nuclear features of malignant tumor cells. After training the algorithm on the “training subset,” these algorithms are prepared to perform GS classifications by segmenting the images according to specific features that it has learned in the training step. The algorithm segments and classifies different tissue regions into benign or malignant areas, further distinguishing between Gleason patterns.

Advanced models, such as U-Net-based FCNs or ResNeXt50-enhanced classifiers, use hierarchical feature extraction to detect subtle architectural variants in tumor growth patterns. These AI-driven systems not only automate the initial scoring process but also quantify tumor heterogeneity, providing quantitative metrics that can assist pathologists in decision-making. Moreover, ensemble learning allows the pooling of information from multiple AI algorithms into a “majority vote,” which can improve AI’s accuracy and robustness of its conclusions. Self-supervised techniques have also been developed, which allow for the algorithm to teach itself the features of images for classification rather than requiring manual labels, which can reduce workload burden and interobserver variability in generating a training dataset.

AI in screening and diagnosis

The FCN with U-Net architecture enhanced by ResNeXt50 represents an advanced deep learning system that is specifically designed for image segmentation tasks such as identifying cancerous regions on WSIs. The system’s U-Net architecture is defined by its “U-shaped” structure that consists of an encoder-decoder design. The encoder’s role is to extract hierarchical features from the input image by progressively compressing the data to capture essential patterns. Feature extraction is the process where a neural network identifies and learns important patterns or characteristics from the data that was input. In this context of prostate cancer, feature extraction would include analyzing WSIs to identify shapes, textures, and arrangements of cells and tissues, which are then used to make predictions or classifications of cancerous regions or grading of Gleason patterns. The decoder’s role is to reconstruct these extracted features to produce high-resolution pixel-level segmentation maps, which ensures precise localization and differentiation of cancerous tissues. A distinguishing feature of the U-Net architecture is its skip connections, which allow for the linkage of layers in the encoder to the corresponding layers in the decoder. This allows the FCN to retain fine-grained spatial details while also simultaneously integrating higher-order contextual information.

The ResNeXt50 is also an architecture that can be included within the U-Net’s encoder, whose role is to enhance feature extraction capabilities by using grouped convolutions. Grouped convolution is a type of operation where input channels are split into smaller groups that are each processed independently, which allows for separate filters to be applied to each group rather than one single convolutional filter being applied across all input channels. The grouped convolutions improve feature extraction efficiency while maintaining computational feasibility [5]. Together, the utilization of the U-Net framework and ResNeXt50 allows for a robust solution for segmenting and analyzing complex histopathological patterns, making it well-suited for Gleason grading in prostate cancer pathology. Another key feature of this model is the integration of atrous spatial pyramid pooling (ASPP), which is added after the encoder-decoder network’s bottleneck. The ASPP is a technique used by CNNs that allows the model to capture contextual information at multiple spatial scales. The ASPP uses an atrous convolution, which applies filters with different dilation rates so it can analyze both small details at the cellular level and larger patterns such as glandular organization. The ASPP then combines all the information from these multiple scales into a single output, which gives the model a better understanding of the image. This process can be especially important for differentiating closely related Gleason patterns, such as grades 3 and 4, where subtle architectural differences can influence treatment decisions. This model also uses ensemble distillation, which can be understood as a “majority vote” of multiple trials. In the original application, five models were trained on different subsets of data, and the aggregate knowledge was transferred to create a student network that improves generalizability and reduces training time [5].

Another model with clinical significance is DeepDx® Prostate, which combines a deep neural network architecture with pixel-level segmentation to analyze prostate core needle biopsies. DeepDx® Prostate processes WSIs in two steps. The first step is patch-level segmentation, where the WSI is divided into smaller cropped regions of fixed size known as patches, which are analyzed individually for five categories ranging from non-cancerous to Gleason pattern 5. The use of patches allows for the reduction of computational demands and the ability to focus on relevant features. The second step is slide-level evaluation, where the results are aggregated into a single heatmap, and the proportion of each Gleason pattern within the heatmap determines the final Gleason score [7]. The model was trained using 1,133 annotated WSIs and subsequently tested on 700 cases for validation. A reference standard was established by pathologist annotations to ensure training data consistency, which is known as supervised learning. DeepDx® Prostate achieved a kappa (κ) of 0.907, indicating substantial concordance with expert uropathologists. The system also demonstrated superior performance in tumor quantification, achieving a correlation coefficient (R) of 0.97 with pathologist-measured tumor lengths compared to an R of 0.90 for original hospital diagnoses [7]. DeepDx® Prostate is useful as a diagnostic and assistive tool for general pathologists by enhancing their efficiency. AI-assisted grading improved concordance with expert Gleason scores from a κ of 0.876 (manual) to a κ of 0.925 while also reducing slide examination time by 34% [4].

Building on the success of clinically validated models such as DeepDx® Prostate, large-scale initiatives such as the PANDA challenge have further explored the potential for broader utilization and refinement of AI for diagnostics, by fostering the development and evaluation of diverse algorithms on an international scale. The PANDA challenge, conducted in 2020, was a large-scale international AI competition organized by a collaboration between Radboud University Medical Center and Karolinska Institute to develop algorithms for the Gleason grading of prostate biopsies. Participants were given a development set containing 10,616 biopsies, divided into 5,160 from Radboud University Medical Center and 5,456 from Karolinska Institute. Additional external validation datasets included 741 cases from the United States and 330 from Europe, and these were given to teams who were selected for the validation phase. Participants created all sorts of models, including CNNs, used ensemble learning techniques, and semi-supervised frameworks were benchmarked against expert uropathologists. Top-performing entries achieved quadratic-weighted κ scores of 0.862 and 0.868 on external validation datasets, which demonstrates the generalizability of AI systems trained on large, heterogeneous datasets [8].

Neural structures and training methods

The U-Net architecture with ResNeXt50 and DeepDx® Prostate both utilize advanced neural structures to achieve high diagnostic precision. As previously explained, the U-Net design excels in pixel-level segmentation tasks, and the ResNeXt50’s grouped convolutions enhance multiscale feature extraction, which improves sensitivity to complex histological features. DeepDx® Prostate uses DeepLab v3+, a neural network designed for detailed image segmentation. It also includes non-local attention mechanisms, which help the model understand relationships between different parts of the image, improving its ability to analyze complex tissue structures [7].

Both of these systems employ advanced learning techniques to teach the models to recognize tumor patterns and predict GS. DeepDx® Prostate relies on supervised learning, where pathologist-labeled datasets are used to train the model. This approach ensures high accuracy and concordance with expert pathologists. However, this approach has its difficulties in the manner that the creation of large, fully labeled datasets is a labor-intensive process for pathologists. Labeling thousands of WSIs with precise GS is labor-intensive, time-consuming, and expensive. Moreover, multiple pathologists have to come to a consensus to establish reliable training sets, which is a resource-intensive endeavor.

In contrast, the U-Net architecture with the ResNeXt50 model utilizes semi-supervised labeling, where partially labeled data with limited annotations is used to supplement the training process. In semi-supervised learning, the algorithm does not require precise human instruction because the algorithm’s training leverages a combination of labeled and unlabeled data, allowing it to learn underlying patterns from the labeled samples while generalizing its knowledge to the larger pool of unlabeled data. This approach reduces the dependency on fully annotated datasets, which makes it a more practical solution. By combining a smaller set of labeled data with a larger pool of unlabeled data, these models can still achieve high performance while easing the labeling burden on pathologists.

Hard-example mining is a technique that can optimize the semi-supervised approach by focusing on learning the most challenging examples with the highest error rate. In this method, the model identifies the most challenging examples, such as those where its predictions deviate significantly from expert annotations, which are then flagged. These cases are then prioritized during training to help the model improve its weakest areas. Focusing on these difficult examples makes the model more skilled at handling subtle and ambiguous patterns in histological images [7].

Transfer learning is another widely used training method that expedites the training process by leveraging knowledge from pre-trained models. Generic datasets such as ImageNet, Microsoft Common Objects in Context, or the Canadian Institute for Advanced Research, which contain millions of annotated images in hundreds of categories, are frequently used as the starting point for training modern prostate cancer detection models. The model can learn general image identification concepts, such as identifying edges, shapes, and objects, due to these datasets. As these pre-trained models already have a basic understanding of visual patterns, they have a substantial advantage when applied to prostate tissue WSIs. Additionally, cross-domain transfer learning improves diagnostic accuracy by bridging gaps across datasets by adapting models trained on one form of cancer (e.g., breast cancer WSIs) to another type (e.g., prostate cancer WSIs) [6].

Outcomes compared to pathologists

DeepDx® Prostate and FCN with U-Net architecture and ResNeXt50 have been shown to outperform standard pathology reports in accuracy and diagnostic consistency. DeepDx® demonstrated a κ value of 0.713 for Gleason grading and 0.922 for overall concordance with expert pathologists, while the original pathology reports demonstrated a κ value of 0.619 for Gleason grading and 0.873 for concordance. The model’s tumor quantification capabilities also exceeded pathologist estimates, highlighting its utility in generating consistent, reproducible diagnostic metrics [4,7]. Similarly, FCN with U-Net architecture and ResNeXt50 also performed well in testing. The system achieved comparable or better performance than individual pathologists composed of both uropathologists and general pathologists, with quadratic-weighted kappa of 0.92, 0.96, and 0.93 on the three test sets compared to 0.65-0.91 for the individual pathologists [5]. Finally, the PANDA challenge, which consisted of a multitude of different AI models, corroborates these findings. On external validation sets, these AI models were able to achieve agreements of quadratic-weighted kappa of 0.862 and 0.868 with expert-level uropathologists [8].

AI for biomarker-based, non-invasive diagnostic approaches

One disadvantage of histopathology-based AI models is their reliance on invasive tissue sampling and its subsequent evaluation. Prostate biopsies entail the insertion of a needle through the rectum or perineum to extract tissue directly from the patient’s prostate gland, which can be costly and invasive to the patient. In contrast, PSA serves as a non-invasive biomarker that can be integrated into AI models to enhance risk stratification. However, elevated PSA levels in the blood are not specific to malignancy. PSA elevation may indicate prostate cancer, benign prostatic hyperplasia (BPH), or prostatitis. As a result, PSA alone is generally considered insufficient for prostate cancer diagnosis as elevated PSA levels can be influenced by benign conditions such as BPH or even recent physical activity such as cycling. Despite PSA’s limitations, AI algorithms that incorporate PSA data remain beneficial due to enhanced stratification of high-risk populations and reduce the need for unnecessary biopsies.

PSA testing is commonly used in prostate cancer screening, serving as a first-line tool for early detection in at-risk populations. By combining PSA levels with AI, models can provide an additional layer of risk stratification, improving early detection while reducing unnecessary biopsies. Perera et al. (2021) highlight one such model to improve prostate cancer risk stratification by using PSA and related biomarkers. Their model utilizes a dense neural network with four fully connected layers that were trained on patient data, including total PSA, free PSA, free-to-total PSA ratio, and age [9]. The training for the model was implied to have been conducted by supervised learning [9]. For optimization, a stochastic gradient descent was employed, a method that improves the model by iteratively adjusting the model’s parameters by minimizing errors through small, incremental adjustments based on computed gradients [9]. It works by calculating the error from small, random samples of data and making small changes to the model based on the calculated error margin. This helps the model learn quickly from large datasets and identify non-linear relationships between biomarkers. The model anticipated a common limitation of AI algorithms called overfitting, where the model becomes too closely tied to the training data and performs poorly on data outside of its training set due to its lack of generalizability. To prevent overfitting, dropout regularization was utilized, which temporarily removes some data points during training to help the model generalize better. Power transformations were used to normalize the input features, making the data easier for the model to process. Finally, bootstrapping was used to create confidence intervals for performance metrics, providing a measure of uncertainty and helping to improve the reliability of the results. Together, these approaches allowed for consistency and improved model performance [9].

The AI model by Perera et al. (2021) was able to demonstrate superior accuracy in predicting prostate cancer compared to traditional PSA-based thresholds. The model was able to achieve an area under the receiving operating characteristic curve (AUC) of 0.72 in the test dataset, which outperformed PSA alone (AUC = 0.63), free PSA (AUC = 0.50), and age (AUC = 0.52). Previously reported findings often use a PSA threshold of 3.0 for prostate cancer diagnosis, which yields a sensitivity of 32.2% and a specificity of 86.7%. In contrast, when the AI model is set to a specificity of 86.7%, its sensitivity improves to 46.4%. Furthermore, the AI model demonstrates powerful performance at high sensitivities, such as when set to 80%, it can achieve a specificity of 45.3%, which reduces unnecessary biopsies and false positives compared to traditional PSA thresholds [9]. Overall, the model’s capability to combine various biomarkers and utilize non-linear decision-making surpassed traditional methods, providing a more refined tool for risk stratification in clinical practice.

MRI and radiological imaging-based prostate cancer management with AI

In addition to biopsied tissue pathology specimens and biomarkers such as PSA, advanced imaging techniques such as multiparametric prostate MRI (mpMRI) can also provide critical insights into tumor aggressiveness and tumor margins. When coupled with AI algorithms, MRI-based prostate cancer management can complement existing clinical workflows and contribute to a more comprehensive and individualized approach to patient care. Conventional mpMRI interpretation faces significant challenges in estimating the full extent of prostate cancer due to its soft tissue resolution in certain areas not matching that of computed tomography (CT) [10]. Inaccurate tumor margin estimation can lead to undertreatment with tumor recurrence or unnecessary excision of benign tissue in surgical margins. Improved precision in locating the tumor margins using AI-assisted interpretation of MRI imaging has the potential to reduce cancer recurrence through clean-margin excision. Additionally, by minimizing the excision of benign tissue with more precise margins, patients can recover with maximally preserved prostate functionality from the remaining benign prostate tissue.

AI algorithms have demonstrated substantial improvements in delineating prostate cancer extent compared to the standard-of-care (SOC) methodologies. Mota et al. (2024) highlighted the impact of AI-assisted MRI analysis in prostate cancer contouring, where AI-derived tumor boundaries exhibited significantly higher accuracy (balanced accuracy of 84.7% vs. 67.2% for SOC) and sensitivity (97.4% for the AI-assisted method vs. 38.2% for SOC). Compared to the SOC of physician-delineated tumor contouring, AI-assisted tumor contouring led to a remarkable increase in negative margin rates (72.8% vs. 1.6%), suggesting its promise in enhancing the precision of focal therapy while reducing overtreatment of non-cancerous tissue [11].

Beyond improving tumor contour estimation, AI models utilizing MRI data have been instrumental in optimizing treatment decisions. AI-assisted contours altered physician decision-making in 28% of cases, shifting treatment recommendations toward more targeted interventions such as focal therapy, while reducing the use of radical prostatectomy in select patients [11]. These findings underscore the role of AI in guiding individualized therapeutic strategies, ensuring that treatment is neither excessive nor inadequate.

Radiomics is an emerging field that focuses on extracting quantitative features of biomedical imaging, called quantitative imaging biomarkers (QIBs), to provide insights into tumor characteristics beyond visual assessment. Texture analysis techniques, such as the gray-level co-occurrence matrix, help capture tumor heterogeneity [12]. Machine learning enhances the accuracy of radiomics in distinguishing benign from malignant lesions and predicting tumor progression. QIBs have also shown potential in monitoring immunotherapy responses for individual patients, revealing specific imaging features that are linked to immune-activated tumor microenvironments [13]. Combining emerging tools such as QIBs with methods such as liquid profiling could facilitate more personalized management of prostate cancer.

Furthermore, AI-enhanced MRI analysis can integrate seamlessly into biopsied pathology specimens and PSA-based AI models to provide a multi-modal assessment of prostate cancer. Biopsy remains the gold standard for histopathologic confirmation, but AI-driven MRI may help guide targeted biopsy strategies, identifying higher-risk tumor regions that may be overlooked. The integration of multimodal AI approaches that span MRI, biopsy specimens, and biomarker-based methods holds promise in refining the clinical care of prostate cancer patients.

Multi-omics-based AI and machine learning in prostate cancer

Genetics often plays an important role in the prognosis of cancer patients. Integrating AI with genomic data can complement other clinical measurements to generate recommendations based on predicted treatment responses. Advanced computational analyses of genomic, epigenetic, and tumor microenvironment data from prostate cancer patients have identified novel biomarkers associated with specific molecular subtypes of prostate cancer [14]. Classification of cancer subtypes based on these molecular features involves utilizing machine learning approaches such as unsupervised clustering algorithms and dimensionality reduction techniques. Computational and bioinformatics analyses have helped identify distinct molecular subtypes of prostate cancer, such as those defined by E26 transformation-specific (ETS) fusion genes, speckle-type POZ protein (SPOP) mutations, and immune-related phenotypes, each with different prognostic implications [14].

Classifiers such as prediction analysis of microarray 50 (PAM50) have also demonstrated their utility in predicting treatment responses in prostate cancer. PAM50 is an algorithm originally developed for breast cancer, using a 50-gene signature to categorize breast cancer into five subtypes, but in Ge et al.’s study, PAM50 was applied to prostate cancer analysis. With these tools, the study identified novel findings about treatment response predictions, including how luminal B subtypes of prostate cancer have a higher response rate to androgen deprivation therapy (ADT) compared to the luminal A and basal subtypes, despite having an overall poorer prognosis [14]. Their findings highlight how advances in computational analyses can be used to optimize treatment types based on the molecular subtype of prostate cancer. Moreover, machine learning algorithms developed for different types of cancer may also be informative for applications in prostate cancer and beyond, as demonstrated by the adaptation of PAM50.

Integration of multiple “omics” datasets, including metabolomics and transcriptomics, has provided an additional dimension to analyze prostate cancer tissues. In metastatic prostate cancer, machine learning models integrating genomic alterations and lipidomic profiles have demonstrated improved prediction accuracy for clinical outcomes [15]. Multi-feature classifiers were used to predict clinical outcomes from genomic and lipidomic data from patients with metastatic hormone-sensitive prostate cancer (mHSPC) and metastatic castration-resistant prostate cancer, which showed AUC scores of 0.751 for predicting mHSPC survival and 0.638 for predicting treatment failure of ADT [15]. Additionally, Ren et al. (2015) demonstrated the capacity of metabolomics tools to uncover altered molecular pathways in prostate cancer tumorigenesis, such as sphingosine-1-phosphate receptor signaling. Their findings indicated a loss of the tumor suppressor gene’s downstream signaling process, which can be targeted for therapeutic interventions in the future [16]. Metabolomics, proteomics, and transcriptomics complement genomics by providing dynamic insights into downstream pathways of genes such as gene expression, protein interactions, and the metabolism of these molecules. This high-resolution data may also be useful in differentiating prostate cancer from similarly presenting benign conditions such as BPH.

AI in prognostic models

Physicians are notoriously inaccurate in estimating the prognosis of patients, especially in predicting survival outcomes for patients with terminal or chronic illnesses [17]. Multiple factors contribute to this phenomenon of prognostic inaccuracy. Some studies suggest that physicians frequently overestimate survival times and provide overly optimistic estimates of survival. In one study, only 20% of prognostic predictions were accurate within 33% of actual survival, with physicians overestimating survival by a factor of 5.3 on average [17]. Prognostic accuracy tends to improve as patients near death, but long-term predictions remain uncertain and highly error-prone. In the case of prostate cancer, clinicians tend to be error-prone in both survival time estimates and their estimates of the benefit of radical treatments such as radical prostatectomy. One study, which compared clinician estimates to Predict Prostate models, found that clinicians’ mortality estimates were more than fivefold higher than model predictions [18]. This error-prone prognostication may have led to overtreatment, such as radical prostatectomy, in patients who may have benefited from a less aggressive treatment course.

Machine learning and AI tools have shown potential in improving the accuracy of prognostic estimates for patients with prostate cancer. Machine learning models such as random survival forests (RSF) and survival trees have demonstrated superior accuracy compared to traditional clinician-based methods in predicting outcomes such as overall survival (OS) and cancer-specific survival (CSS) in metastatic prostate cancer [19]. For instance, RSF models achieved higher predictive accuracy (C-index up to 0.832) compared to conventional Cox regression models or PSA-based predictions [19]. These studies demonstrate that machine learning algorithms are capable of accounting for the non-linear and combined impact of multiple features of a disease course. The increased accuracy of these models compared to clinician estimates may be attributable to this capacity to systematically integrate complex, multifactorial variables into mathematical algorithms.

## Discussion

AI-driven systems have shown the potential to improve the GS process by enhancing diagnostic consistency and accuracy. The traditional GS, which assesses tumor architectural patterns, is prone to inter- and intra-observer variability among pathologists [4]. AI models such as FCNs with U-Net architecture enhanced by ResNeXt50 and DeepDx® Prostate mitigate these inconsistencies by analyzing WSIs and providing standardized, quantitative evaluations [5,6]. These AI systems use multi-step pipelines, starting with WSI digitization, preprocessing techniques such as stain normalization and data augmentation, and advanced deep learning models for feature extraction. AI-based segmentation and classification improve pathologist workflow efficiency, with ensemble learning techniques further refining predictive accuracy [5].

The DeepDx® Prostate model, the first clinically approved AI system for prostate cancer diagnosis, has demonstrated high concordance with expert pathologists and even demonstrates improved diagnostic efficiency, although it is highly time and labor-intensive to develop this type of supervised learning model [4,7]. Further, top entries for the PANDAS challenge in 2020 demonstrated the generalizability of AI systems trained on large, heterogeneous datasets [8]. Semi-supervised learning models, such as the U-Net architecture with ResNeXt50, reduce the dependency on fully annotated datasets while still achieving high performance. This practical approach can be optimized by hard-example mining, in which examples with the highest error rate are prioritized during training for targeted model improvement [7]. Furthermore, transfer learning can leverage pre-trained models with cross-domain transfer learning to improve the diagnostic accuracy of one type of cancer to another [6].

Integration of AI with other diagnostic and prognostic tools, including PSA testing, MRI-based imaging, and multi-omics approaches [9,20]. AI-assisted MRI analysis in prostate cancer contouring showed higher accuracy and remarkably higher negative margin rates compared to SOC methodology [11]. Radiomics incorporates machine learning techniques to extract quantitative imaging biomarkers for tumor classification and monitoring treatment responses. Multi-omics AI models analyze genomics. Metabolomic and transcriptomic data can be used to identify promising personalized treatment strategies [15,16]. AI-driven prognostic models outperform clinician estimates to predict survival outcomes, mitigate biases, and optimize treatment decisions [19]. Collectively, these advancements demonstrate AI’s potential to refine prostate cancer diagnosis, personalized approaches to patient management, and improve prognostic accuracy while simultaneously reducing healthcare inefficiencies.

While there have been very promising developments of AI to aid in the diagnosis and prognosis of prostate cancer, there are challenges associated with using these AI systems. AI models are susceptible to biases introduced by non-representative training datasets, leading to disparities in diagnostic accuracy among diverse populations. For instance, the systematic review by Frewing et al. highlighted observer variability in pathologist annotations as a source of bias, emphasizing the need for more inclusive datasets [6]. Domain shift bias, caused by differences in imaging protocols across institutions, poses a significant challenge and highlights the need for universal stain normalization [9]. Coupled with small and heterogeneous datasets, domain shift bias constrains the robustness of AI models. This was demonstrated by the PANDA challenge, in which even the best-performing models struggled with the diagnosis of benign cases in external datasets due to dataset-specific domain shifts [8]. Given these ongoing challenges, generalization of these AI models remains a hurdle, where models may perform very well in controlled settings but struggle in real-world clinical environments [20].

## Conclusions

Overall, AI models such as U-Net architecture with ResNeXt50 and clinically validated systems such as DeepDx® Prostate demonstrate high diagnostic precision and perform better than standard pathology reports in both tumor detection and Gleason grading accuracy. Integrating PSA data with these neural networks showed superior accuracy in risk stratification compared to traditional PSA thresholds, which can decrease unnecessary prostate biopsies and healthcare utilization. Importantly, addressing limitations such as data heterogeneity, domain shifts, and the ongoing need for stain normalization can improve the generalizability of these models, thereby revolutionizing their role in prostate cancer diagnosis and improving clinical efficiency.
